# Combining Computational Prediction of *Cis*-Regulatory Elements with a New Enhancer Assay to Efficiently Label Neuronal Structures in the Medaka Fish

**DOI:** 10.1371/journal.pone.0019747

**Published:** 2011-05-27

**Authors:** Emmanuel Mongin, Thomas O. Auer, Franck Bourrat, Franziska Gruhl, Ken Dewar, Mathieu Blanchette, Joachim Wittbrodt, Laurence Ettwiller

**Affiliations:** 1 McGill Centre for Bioinformatics, McGill University, Montréal, Canada; 2 Centre for Organismal Studies COS, University of Heidelberg, Heidelberg, Germany; 3 MSNC INRA Group, UPR2197 DEPSN Institut Fessard, CNRS, Gif-sur-Yvette, France; 4 McGill University and Genome Quebec Innovation Centre, Montreal, Canada; 5 Karlsruhe Institute for Technology KIT, Institute for Toxicology and Genetics, Eggenstein-Leopoldshafen, Germany; National University of Singapore, Singapore

## Abstract

The developing vertebrate nervous system contains a remarkable array of neural cells organized into complex, evolutionarily conserved structures. The labeling of living cells in these structures is key for the understanding of brain development and function, yet the generation of stable lines expressing reporter genes in specific spatio-temporal patterns remains a limiting step. In this study we present a fast and reliable pipeline to efficiently generate a set of stable lines expressing a reporter gene in multiple neuronal structures in the developing nervous system in medaka. The pipeline combines both the accurate computational genome-wide prediction of neuronal specific *cis*-regulatory modules (CRMs) and a newly developed experimental setup to rapidly obtain transgenic lines in a cost-effective and highly reproducible manner. 95% of the CRMs tested in our experimental setup show enhancer activity in various and numerous neuronal structures belonging to all major brain subdivisions. This pipeline represents a significant step towards the dissection of embryonic neuronal development in vertebrates.

## Introduction

Recent years are witnessing a flood of new discoveries in neuroscience largely resulting from the ability to monitor living cells in the context of the developing nervous system using reporter gene expression [1]. Exciting development in engineering new proteins has extended current barriers to allow monitoring and manipulating the activity of specific pathways within living cells [2]–[5]. Nonetheless, these techniques rely heavily on the ability to drive gene expression to specific developmental stages, brain structures and cell types in a stable and reproducible way. While great efforts have been made to efficiently obtain such stable lines, this step remains a serious bottleneck.

In vertebrates, the most widely used strategy to express reporters in anatomical structures relies on the use of regulatory elements, often promoters of genes known to be expressed in the desired structures (promoter bashing). This trial and error process is slow and tedious. Thus, to maximize the chances of getting the right regulatory sequences, entire loci around selected genes employing BAC technology have been used [6]. However, this methodology is time-consuming and the level of reporter expression may not be high enough for proper monitoring. Other attempts to generate reporter gene expression in various structures are based on the random insertion of a reporter cassette into the genome [7]–[11]. Only upon activation by nearby regulatory element(s), the transgene is expressed (enhancer trap). In mouse [12] and zebrafish [13],[14], enhancer assays have been developed essentially to test genomic elements for enhancer activity.

Despite advantages of one approach over the other, all these methodologies have the significant drawback of lacking specificity. Testing semi-random elements in vertebrates either by promoter bashing or enhancer traps results in high screening efforts, while BAC technology, which addresses the specificity issue by using the entire locus instead, is experimentally tedious and cannot be scaled up easily.

In parallel, progress has been made towards the computational identification of regulatory regions in sequenced genomes. Previous work has shown that, without experimental priors, functional constraints acting on non-coding sequences are one of the most predictive information to locate regulatory elements [15],[16]. Thus cross-species comparison has been extensively used to improve the detection of functional non-coding DNA regions from neutrally evolving DNA [17]. The discovery of new regulatory regions using inter-species conservation was greatly stimulated by the recent availability of various vertebrate genomes, from mammals to fish [18]–[21] as well as the development of more specific and sensitive alignment programs [22]–[25]. Furthermore, it has been shown that the tendency of transcription factor binding sites (TFBS) to cluster together can be used to predict putative CRMs [26]. This led to the development of new methods to locate clusters of binding sites in conserved regions [27]. An algorithm that combines both, inter-species binding site conservation and clustering has recently been applied to the human genome [28] resulting in the identification of 118,000 predicted human regulatory elements [29].

**Figure 1:**
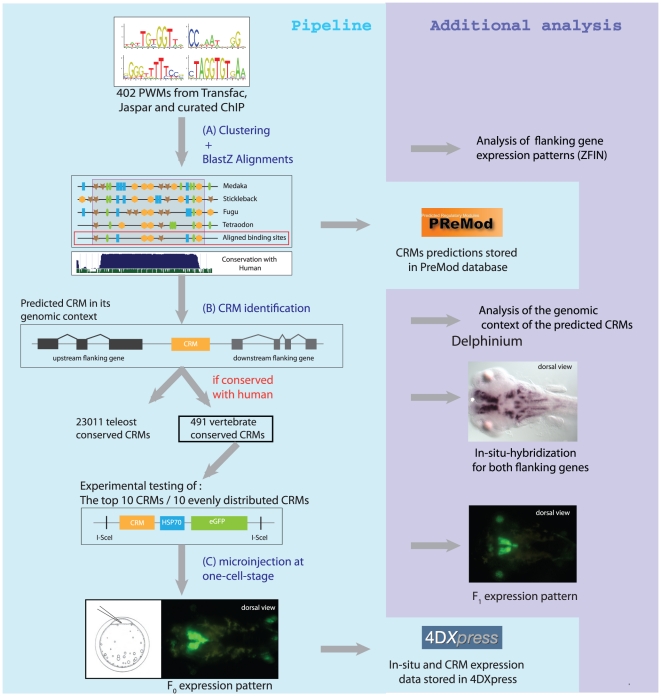
Schema of the pipeline. (**a**) and (**b**) correspond to the computational prediction of CRMs. A subset of these CRMs is then experimentally tested *in-vivo* (**c**) and the results of the computational prediction and experimental analysis are stored in two databases, PreMod and 4DXPress, respectively. To evaluate the pipeline, additional experiments were performed (location analysis, analysis of the flanking gene expression patterns for -1- the experimentally validated CRMs (by whole mount *in-situ* hybridization) and -2- all predicted CRMs (using ZFIN annotations)).

Here, we report the development of a new pipeline aimed at specifically labeling, in a stable manner, various neuronal structures in developing *Oryzias latipes* (medaka) embryos. This pipeline represents two major breakthroughs compared to previous methodologies: A selective step to predict neuronal specific regulatory regions, combined with a new reliable enhancer assay in medaka to efficiently obtain stable lines expressing the reporter gene in neuronal structures (**Figure 1**).

The selective step applies a modified version of the computational pipeline previously described [28] to select a large number of short (∼100–1000 bp) regions predicted to be CRMs in fish. As we predict vertebrate conservation to be an important criteria for selecting CRMs active in neuronal structure, we filtered those regions conserved until human and tested them in our new enhancer assay in the medaka fish. As expected, a vast majority of the regions resulted in a strong, reproducible expression of the reporter gene in various neuronal structures. All the major subdivisions of the medaka CNS were covered by at least one expression pattern. In most of the cases, the reporter gene expression persists beyond hatching and in all cases analyzed, at least two independent stable lines were generated. We also show that the enhancer activity is reminiscent of the endogenous target gene expression, which facilitates the additional selection of regions to target specific anatomical areas. Both, the computational prediction of CRMs and the experimental results have been integrated into databases for easy access and queries.

Taken together, our pipeline is an important tool for labeling neuronal structures and deciphering the regulatory grammar controlling the development of the neuronal system in vertebrates. Furthermore our results indicate that pan-vertebrate conserved non-coding elements compared to less deeply conserved elements, show activity preferentially in neuronal structures.

## Results

### Identification of a set of neuronal regulatory elements

One of the key steps to establish a robust pipeline for the labeling of developmental structures is the accurate prediction of autonomous regulatory elements in the genome. Thus, to define genomic regions most likely involved in gene regulation, we use a variant of the PreMod algorithm [28] applied to the medaka genome (see Methods). The algorithm first identifies individual TFBS based on a set of 402 high quality position-weight matrices (PWMs), from manually curated databases of known TFBS (Transfac [30], Jaspar [31]) and results from ChIP data [32] (**Figure 1** **and** **methods**). Next, it assesses conservation of the predicted TFBS by comparing the medaka sequence to the orthologous sequences in *Tetraodon nigroviridis* (tetraodon), *Takifugu rubripes* (pufferfish) and *Gasterosteus aculeatus* (stickleback). Finally, clusters of conserved homotypic or oligotypic binding sites were identified and predicted as CRMs (**Figure 1**).

The algorithm resulted in the identification of 23,011 predicted CRMs (average length 244 bp; median length 136 bp) which contain on average 62 putative TFBSs. These regions, despite being broadly distributed over the genome, are found significantly more often in intergenic regions (72.4%, p-value <0.01, **Figure S1A**) and preferentially within 100 kilobases (kb) distance to the nearest transcription start site (TSS) (93.11%, p-value <0.01, **Figure S1B**).

It has previously been shown that vertebrate conserved non-coding elements are functional enhancers [12]. These elements are also known to be preferentially located around developmental genes and are consequently hypothesized to be active during development [16]. Thus, we selected those predicted CRMs for which a statistically significant alignment in a conserved syntenic block with human was found (see Methods for details). Of the resulting 491 vertebrate conserved CRMs, 69.36% lie in intergenic regions (p-value <0.01, **Figure S1A**) and 97.98% are located less than 100 kb away from the nearest TSS (p-value <0.01, **Figure S1B**). These trends are accentuated compared to the ones observed for the entire set of predicted CRMs.

Both sets of predicted CRMs (all CRMs and vertebrate conserved CRMs) are stored in the PreMod database [29] (http://premod.mcb.mcgill.ca) and listed in Supplementary Tables S1 and S2. PreMod provides the location, score, and binding site content of each predicted CRM. It also reports which transcription factor matrices were used to build the CRM (tag matrices). Predicted CRMs and surrounding genes are displayed in their genomic context. Where *in-situ* expression of medaka genes or CRM activity information is available, PreMod links to the corresponding experimental data stored in the 4DXpress database [33] (http://4dx.embl.de/4DXembl/reg/all/searchbyspecies/line.do?speciesID=4).

**Figure 2:**
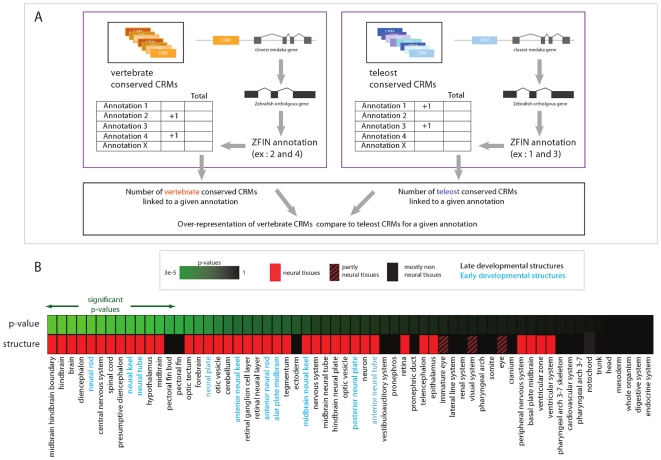
Analysis of the genomic location of CRMs. A. Schema of the procedure. For each predicted CRM, the closest medaka gene is identified. Next we transferred zebrafish *in-situ* annotation to the medaka orthologous gene. We calculated the significance of the overrepresentation of CRMs showing annotations for specific tissues from the vertebrate conserved dataset compared to a background set (composed of the whole set of predicted CRMs). B. Enrichment of vertebrate conserved CRMs around genes expressed in neuronal tissues. Red squares correspond to neuronal structures. P-values are shown with a color code, the most significant enrichments correspond to the p-values in green, the least significant to p-values in black. Significant p-value cutoff has been determined for a 5% false discovery rate (Benjamini, Hochberg method, see **Supplementary Table S4** for numerical values).

Next, we took advantage of the large compendium of *Danio rerio* (zebrafish) *in-situ* annotations from ZFIN [34] to shed light on the putative function of the predicted CRMs. We first mapped the *in-situ* annotation of the zebrafish genes onto their orthologs in medaka (**Methods** **and** **Figure 2A**). For each of those predicted CRMs in the medaka genome, we located the closest of the two flanking genes and assigned its projected ZFIN annotation to the CRM. We then tested if vertebrate conserved CRMs show a statistically significant increase in annotations for certain developmental tissues compared to the rest of predicted CRMs. Interestingly, we found that vertebrate conserved CRMs are associated with an elevated ratio of genes expressed in various brain regions compared to all predicted CRMs (**Figure 2B**; **Tables S3 and S4**). More specifically, 74% of vertebrate conserved CRMs are associated with genes annotated as being expressed in the central nervous system (brain: p-value  = 5e^−4^,spinal cord: p-value  = 2e^−3^). On the other hand, enrichment is not observed in non-neuronal tissues (pronephros: p-value  = 0.22, somite: p-value  = 0.45, cardiovascular system: p-value  = 0.67).

This finding, empirically observed in mouse enhancer analysis [12] and confirmed in this study, has important implications for the understanding of neuronal system evolution in vertebrates. Vertebrate conservation can be used as criteria to prioritize which regulatory elements to use for the labeling of neuronal structures.

### Development of a new enhancer assay in medaka

We developed a new enhancer assay to rapidly test genomic regions for enhancer activity and to derive stable transgenic lines. Aiming to set up a pipeline for large-scale analysis, we particularly focused on generating a quick and reliable readout, which required live monitoring of the expression pattern directly in injected embryos. The ability to record GFP expression in a live embryo throughout its development is a clear advantage of the fish system compared to the mouse embryo. Thus, we expect an increased sensitivity in the detection of expression patterns and better characterization of these expression patterns over time.

We use meganuclease mediated transgenesis [35] as the method of choice to obtain highly efficient integration of the transgene into the genome and high rates of germline transmission. Predicted CRMs are cloned into a pBlueScript-based transgenesis vector containing two recognition sites for the meganuclease ISce-I [36] flanking a core promoter, a reporter gene and a SV40-polyadenylation signal. Injected embryos were visually monitored daily for a week to follow the spatio-temporal pattern of GFP expression during embryonic developmental stages (**Figure 1**).

We also developed a robust and efficient experimental setup to distinguish between the absence of enhancer activity and the failure of the injection experiment. For this, we use the hsp70 core promoter that conveniently triggers a strong and specific lens expression from stage 28 onward [37]. The heat-inducible zebrafish hsp70 gene is expressed during normal lens development under non-stress conditions. This feature remains when CRMs are cloned upstream of the core promoter, resulting in embryos with composite expression in the lens and other domain(s) (if any) specific for the CRM. As the correlation between lens expression and expression in other domains is very high when testing positive CRMs, the monitoring of lens expression itself is a very good indicator for the injection success rate.

We therefore monitor the number of lens-positive embryos (injection success rate) and the number of embryos showing reproducible GFP expression in other domains (**Table S5**). The percentage of successfully injected embryos showing reproducible expression outside the lens is calculated and should be above 50% in order to call a genomic region positive for enhancer activity. To be significant, a consistent pattern should be seen for at least 10 individual fish. This typically requires injecting less than a hundred embryos, which is easily achievable in a single injection experiment. About 1 in every 50 successfully injected embryos shows non-consistent expression most likely resulting from the activity of local enhancers (enhancer trap). Following our defined criteria, the enhancer trap expression pattern does not pass the quality control and is therefore discarded. This quality control measurement is a significant improvement over previously described enhancer assays from which the distinction between injection failure and lack of enhancer activity cannot be made.

In a typical experiment we obtain an injection success rate around 46%, and, in the case of a functional enhancer, on average 66% of successfully injected embryos show a consistent expression pattern (**Table S5**). These highly reproducible patterns are a good indication that the expression patterns we observe are solely the result of the tested enhancer activity.

### A vast majority of the computationally predicted regions shows enhancer activity

The top 10 computationally predicted vertebrate CRMs located in eight genomic loci were experimentally tested for enhancer activity and the injected fish were raised to generate stable transgenic lines (**Table S6a**). To evaluate the global success rate of the pipeline, an additional 10 predicted CRMs evenly distributed among the 200 top scoring candidates were tested for enhancer activity (**Table S6b**).

To ensure the inclusion of all the necessary regulatory features, we fused close-by predicted CRMs (see Methods) and extended the predicted regions to include 200 bp flanking sequence on each side. The resulting regions are ranging from around 500 bp to 2 kb and their location varies from 2095 bp to 63755 bp distance to the TSS of the nearest gene (20 kb on average).

**Figure 3:**
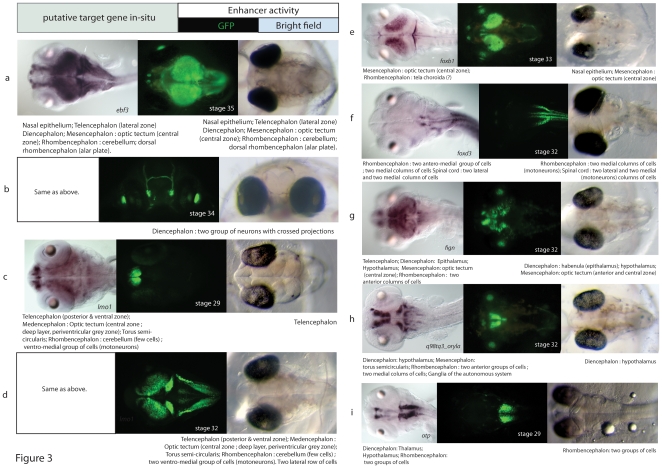
Summary of the expression patterns in stable lines. (a–i) *In-situ* hybridization of the flanking gene (left) and stable lines expressing GFP under the control of the corresponding CRM (right). MEDMOD046561 did not show detectable enhancer activity. For more details on the expression patterns of the transgenic lines, see Supplementary Figures 5–9. All embryos are shown in a dorsal view unless stated otherwise. (**a**) MEDMOD021953, (**b**) MEDMOD021885 (frontal view), (**c**) MEDMOD062537, (**d**) MEDMOD062451, (**e**) MEDMOD074008, (**f**) MEDMOD070042, (**g**) MEDMOD046007, (**h**) MEDMOD045693, (**i**) MEDMOD086628.

Out of the 20 tested regions, 19 triggered a reproducible expression pattern in transient transgenic fish (**Figure 3**, **Figures S2**, **S3**, **S4**, **S5**, **S6**, **S7**, **S8**, **S9**). Extrapolated to the full dataset of the 200 top scoring regions, we estimate that 95% of the computationally predicted CRMs have enhancer activity during embryonic development. The fraction of validated enhancers is higher than for another large-scale study done in mouse, which reveals that 40% of ultra-conserved elements show enhancer activity [12]. This result is further discussed but may be caused by both, the prediction method involving vertebrate conserved regions and the monitoring of reporter gene expression throughout the whole embryonic development.

Stable transgenic lines were generated for all the top nine candidate regions with validated enhancer activity. The same spatio-temporal structures were labeled in transient injected fish compared to stable lines showing that the accurate description of enhancer activity can be done directly in the injected fish. Thus, the required experimental time can be cut down from eight weeks (generation time of medaka) to less than a week (time for embryogenesis in medaka).

### Stable expression of the reporter gene in neuronal structures

Further confirming the computational predictions, all the positive elements drive reporter gene expression in various neuronal structures. Some patterns are limited to very specific areas of the brain or the peripheral nervous system, sometimes, with just a few cells being labeled. This specific expression remains in stable lines suggesting that the reporter gene expression is activated in only one or a few cell types. For example, MEDMOD021885 highlights a cluster of a few dozen neurons located bilaterally in the diencephalon (**Figure 3d**). Other CRMs gave broader expression patterns, covering entire domain(s) of the brain.

For a general analysis of the neuronal system, a complete coverage of brain structures would be desired. We found that all major subdivisions of the vertebrate CNS include labeled cells in our assay. Reporter gene expression is found in telencephalic domains (line MEDMOD021953), the diencephalon (lines MEDMOD021953, MEDMOD021885, MEDMOD046007), the mesencephalon (lines MEDMOD074008, MEDMOD021953), the rhombencephalon (lines MEDMOD021953 and MEDMOD070042, among others), and the spinal cord (line MEDMOD070042). Other neuron-containing structures, such as the nasal epithelium were also labeled (lines MEDMOD21953 and MEDMOD074008) (**Figure 3**; **Figure S2**).

The expression patterns of the lines have been annotated using a controlled vocabulary from the medaka anatomical ontology [38] and incorporated into 4DXpress. From the 32 defined neuronal structures in the ontology, 20 (62%) were labeled in at least one of the stable lines generated. These stable lines expressing a reporter gene in specific cell types are an important starting point for further functional analysis of defined brain structures. In the long run, they offer a valuable resource for the accurate characterization of neuronal cell types and the anatomical description of embryonic neural structures in vertebrates.

Next, we investigated whether the reporter gene expression monitored in our stable lines reflects the expression pattern of the genes surrounding the CRMs in their native genomic location. For this we performed whole-mount *in-situ* hybridization of the genes flanking the CRMs and compared the resulting expression patterns with the activity of the enhancers (**Figure 1**). For each of the nine predicted CRMs showing enhancer activity, we found that at least one of the flanking genes is expressed during development (**Figure 3**). Furthermore, at least one spatio-temporal domain of expression is common with the reporter gene expression under the control of the corresponding enhancer. These results strongly suggest that our enhancer assay outputs represent an accurate description of the activity of the enhancers in their native endogenous state.

The algorithm defines a list of transcription factors predicted to bind to the predicted CRMs. To evaluate how pertinent this information is, we selected three experimentally confirmed CRMs whose activity is restricted to a very defined neuronal structure (forebrain, diencephalon or rhombomeres). Using the ZFIN database, we compared the expression pattern of the factors predicted to bind these CRMs to the observed enhancer activity and searched the literature for those transcription factors being expressed in overlapping domains (**Table S7**).

For the CRM active in the rhombomeres (MEDMOD086628) we found, among others, following transcription factors: MafB (Val), known to be required for hindbrain segmentation and rhombomere formation [39], Elf1 that belongs to the ephrin family which is involved in rhombomere boundary specification in zebrafish [40] and Evi1 that has been shown to be expressed in rhombomeres. Interestingly, Evi1 is a target gene of the MafB repressed transcription factor gene, hoxb1a [41],[42]. These three transcription factors (MafB, Elf1 and Evi1) have all been predicted to bind the MEDMOD086628 CRM, but expression domains of Elf1 and Evi1 are not limited to the rhombomeres. Only MafB is preferentially expressed in the rhomobomeres suggesting that MafB restricts the CRM activity to this structure.

For the CRM active in the diencephalon (MEDMOD045693), four transcription factors are predicted to bind this CRM (Pou3f2, Hnf6, dl and Fos) and show overlapping and specific expression patterns. Pou3f2, for example, is required for oxytocin neuronal development in the hypothalamus [43]. All these factors are expressed in additional domains suggesting that the coordinated action of these factors in the telencephalic domain is required for the CRM activity. The same holds true for the forebrain CRM (MEDMOD062537).

Taken together, these results show that the factors predicted to bind these CRMs can be used as starting points to prioritize further experiments.

## Discussion

We describe a new hybrid methodology aimed at identifying neuronal regulatory elements in fish. With 95% success rate after experimental validation and a 100% success rate in transgenesis, this pipeline is, to date, the most efficient procedure to obtain stable transgenic lines expressing reporter genes in various neuronal structures. Furthermore, the orthologs of three of the 20 CRMs analyzed in our study have previously been tested in mouse [12]. For one of the sequences assayed (homologous to MEDMOD021953), expression of the reporter gene localized to the hindbrain of mouse at stage E11.5. In comparison, MEDMOD021953 also shows expression in the medaka hindbrain but is not restricted to this structure. No expression was observed for the other mouse sequence assayed by Pennacchio et *al*. [12] (homologous to MEDMOD086628) while it drives reporter gene expression in the rhombomeres in our study. These results indicate the high sensitivity of the enhancer assay in medaka.

We have also shown that the patterns of reporter gene expression in our lines are reminiscent of the expression of genes neighboring the tested CRMs. Using gene expression information such as *in-situ* data, it will therefore be possible to further target the pipeline to select regions most likely active in specific neuronal structures. This task is facilitated by the fact that the computational predictions stored in PreMod are linked to expression data stored in 4DXpress. Furthermore PreMod provides CRMs in their genomic context as well as a score for each predicted regulatory region. As a result, prior to *in-vivo* testing, CRMs can be targeted based on their genomic context and score.

Finally, we have shown that the predicted CRMs conserved across vertebrates are enriched around genes known to be expressed in neuronal tissues. Such enrichment cannot be detected for non-neuronal tissues (with the notable exception of pectoral fin and pectoral fin bud) suggesting that this trend is essentially neuronal specific. This analysis, (supported by the experimental results) indicates that pan-vertebrate conserved CRMs have preferred activity in neuronal structures. Our results are in accordance with a recent finding reporting that a large population of heart enhancers is poorly conserved [44] and suggests that the evolutionary conservation of enhancers can vary depending on tissue type. Conservation may reflect the ‘ancestrality’ of neuronal structures but could also reflect the tendency of alignment algorithms to perform better when co-linearity is preserved. Future analysis of such conservation will shed light on evolutionary events that lead to morphological innovation via the emergence of new regulatory interactions.

Our pipeline, designed to create neuronal tissue specific markers, is of great interest for analyzing enhancer activity, identifying genetic markers and finally as a cost effective enhancer screening tool.

## Methods

### CRM prediction

We collected a comprehensive set of 402 non-redundant PWMs based on Transfac (version 9.2) [30], Jaspar core vertebrate matrices [31] and a curated set of matrices built from Chip data with Trawler [32]. Transfac matrices were filtered based on the following rules:

- All non-vertebrate transfac matrices were removed, except for 8 drosophila matrices for factors known to be involved in vertebrate development;
- Matrices linked to more than two different TFs (from the same species) were discarded;
- Among different matrices for the same TF, only that with the highest quality value was kept or, if not available, the predicted sites that are the most conserved through vertebrate evolution were used (M. Blanchette, unpublished).

For each TF, binding sites were predicted in the complete non-coding and non-repetitive regions of euteleostei (based on Ensembl database version 41 [45] of medaka (*Oryzias latipes*, assembly HdrR, Oct 2005 [46]), tetraodon (*Tetraodon nigroviridis*, assembly, Tetraodon 7, Apr 2003 [47]), stickelback (*Gasterosteus aculeatus*, assembly Broad S1, Feb 2006, Broad Institute) and takifugu (*Takifugu rubripes*, assembly 1.0, Aug 2002 [21]) genomes). We followed the procedure described in [28], with the following modifications:

- The local GC-content background model used in [28] was replaced by a uniform background model;
- Interspecies binding site conservation was measured using a more fexible approach that allows for (but penalizes) sites that are slightly misaligned, up to 20 bp. In addition, conservation was weighted as follows: hitScorealn(m, p) =  hitScoremedaka+max(hitScoreTetraodon, hitScoreStickleback, hitScoreFugu). hitScore will then depend on both the score of the binding site in medaka and its conservation in at least one other teleost. Note that a binding site can have a high score without being conserved if the medaka scoring hit is strong enough. CRMs are predicted genome-wide and are not targeted to specific regions (regions with known developmental genes for example).

A subset of 491 CRM predictions was selected using criterion combining high CRM score and conservation with human (vertebrate conserved CRMs). Specifically, predicted CRMs with a BLASTZ [23] score over 2600 between medaka and human and with a percentage identity over 60% were ordered in descending order of CRM scores. BLASTZ homology searches in human were restricted to the orthologous neighborhood of each CRM, defined as following: Each medaka CRM was first associated to the closest medaka gene having a human ortholog H, and the human genes flanking H on the left and the right were identified. From the list of vertebrate conserved CRMs, we selected two datasets: [1] The top 10 scoring CRMs and [2] 10 CRMs distributed at regular intervals in the top 200 scoring CRMs (CRM at position 20, 40, 60, 81, 100, 120, 140, 159, 180, 200) for experimental validation.

### Gene expression analysis

Each predicted CRM is associated with the closest gene independently of the genomic distance between them. We took advantage of the large collection of genes with zebrafish *in-situ* annotations available from the ZFIN *in-situ* database [34]. Next, we transferred zebrafish *in-situ* annotation to the medaka orthologs using the BioMart utility [45],[48]. If more than one ortholog was found for a given zebrafish gene, the orthologous gene with the highest identity was used. For each tissue (and its subparts) and stage, we retrieved all expressed genes. The expression annotation of each gene was subsequently transferred to the associated CRMs (**Table S3**). Only tissues associated with at least 20 vertebrate conserved CRMs are retained for further analysis. We then calculated the significance of the overrepresentation of CRMs showing annotation for specific tissues comparing the vertebrate conserved dataset to a background set (composed of the whole set of predicted CRMs, except vertebrate conserved). The significance of this overrepresentation was calculated with a one-sided fisher test. All tissue and stage annotations follow the OBO ontology.

### CRM genomic location analysis

For each CRM, the distance to the nearest annotated TSS (as defined in Ensembl version 61) is retrieved and categorized into distances of less than 1 kb, 1 to 10 kb, 10 to 100 kb or more than 100 kb. We also assessed if the CRMs are localized in annotated genes or in intergenic regions (<100 or >100 kb away from the nearest gene as defined in Ensembl version 61). One hundred randomizations consisting of the same number of random locations (with the same size distribution) in the medaka genome as the number of CRMs in the real dataset has been produced. The same location analysis was then performed on these random datasets and the significance was calculated from these randomizations.

### Molecular cloning

The identified CRMs were PCR amplified (using LA-Taq polymerase, Takara Bio Inc.) from genomic medaka DNA and flanking HindIII restriction sites introduced (for primer sequences see **Table S8**). After restriction digest the fragments were cloned into a pBlueScript-based transgenesis vector containing two recognition sites for the meganuclease ISce-I [35] flanking a multiple cloning site followed by the core promoter hsp70::GFP [37] and an SV40 polyadenylation signal (clone available upon request). All constructs were verified by sequencing.

### Medaka injection and screening

Injections were done as described [49]. DNA was purified using the Maxiprep Kit (Qiagen) and injected at a concentration of 15 ng/µl.

A Leica fluorescent microscope (Leica MZFLIII) was used to examine GFP expression in live embryos. Injected embryos were analyzed at different stages to determine the spatio-temporal pattern of GFP expression. As the hsp70 core promoter is activated by temperature changes, the embryos were kept and examined at constant room temperature. Developmental stages were determined by morphological features as described by Iwamatsu [50].

### Whole mount *in-situ* hybridization

For analysis of *scamp1*, *fign*(1 of 2), *atg4c*, *gon3_oryla* and *kcnh7* expression patterns, fragments were PCR amplified from medaka cDNA (using Taq-Polymerase, primer sequences in **Table S8**) and subcloned using the TOPO TA Cloning Kit (Invitrogen). After verification by sequencing, Digoxigenin incorporated antisense-RNA probes were generated by *in-vitro* transcription with Sp6 or T7 RNA Polymerase (NEB).

Probe preparation and whole mount *in-situ* hybridization were performed as described previously [51]. For the remaining genes analyzed, we could find at least one clone matching part of the transcript sequence in our in-house library (in pCMV-Sport6.1). In these cases, probes were generated by *in-vitro* transcription with Sp6 or T7 RNA Polymerase directly from these clones.

### Medaka annotation

The medaka nervous system ontology is derived from the medaka fish anatomy and development OBO ontology (medaka_ontology.obo). The descendent terms of nervous system at various stages were extracted. A total of 32 different terms were found and used for the controlled vocabulary annotation. Reporter gene expression was found in 20 (62%) of these anatomical terms.

## Supporting Information

Figure S1 A. Location of the predicted CRMs relative to genes in the medaka genome. The percentage of intragenic, intergenic (<100 kb) and intergenic (>100 kb) locations for all the 23,011 predicted CRMs (left), the 491 vertebrate conserved CRMs (center) and 23,011 random positions in the genome (right) is calculated. The distribution of the CRMs in each category is significantly different compared to random locations, with more CRMs being intergenic (>100 kb). B. Locations of the predicted CRMs relative to the nearest annotated TSS in the medaka genome. For all the 23,011 predicted CRMs (left), the 491 vertebrate conserved CRMs (center) and 23,011 random positions in the genome (right) the distance to the nearest TSS is calculated and binned into less than 1, 1–10, 10–100 and more than 100 kb windows. The percentage of regions for each bin is then calculated. The distribution of the CRMs in each bin is significantly different compared to random locations, with more CRMs being closer to the nearest TSS than expected.
(TXT)

Figure S2 Enhancer activity of the additional 10 predicted CRMs evenly distributed among the 200 top scoring candidates. Example of injected fish showing a reproducible expression pattern. **(a)** MEDMOD021445 **(b)** MEDMOD092210 **(c)** MEDMOD062490 **(d)** MEDMOD057815 **(e)** MEDMOD021442 **(f)** MEDMOD093196 **(g)** MEDMOD062408 **(h)** MEDMOD047799 **(i)** MEDMOD083481 **(j)** MEDMOD062206.
(TXT)

Figure S3 Stable lines at various developmental stages and views for MEDMOD021953. Stage 26–30: Telencephalon, retina ganglion cells (RGCs), tectum central zone, cerebellum, hypothalamus, rombomeres, alar plate. Stage 33–35: Telencephalon, tectum central zone, hypothalamus, cerebellum, hindbrain, RGCs, tegmentum.
(TXT)

Figure S4 Stable lines at various developmental stages and views for MEDMOD062451. Stage 27: One group of cells in the hypothalamus (bilateral) or tegmentum. Stage 32: Optic tectum differentiated cells (central zone), torsus semicircularis, few cells in cerebellum, lateral part of the myelencephalon, hindbrain: two lateral rows cells and two medial rows of cells (motor neurons). Stage 35: Telencephalon posterior (area ventro-posterior), optic tectum: differentiated cells (peri-ventricular grey zone, deap layer), torsus semicircularis, midbrain dorsal, a few cells in the cerebellum anlage, hindbrain: Two lateral rows cells and two medial rows of cells (motoneurons).
(TXT)

Figure S5 Stable lines at various developmental stages and views for MEDMOD074008 and MEDMOD021885. MEDMOD074008 stages 28–37: Tectum central zone and olfactory pits. MEDMOD021885 stages 34–35: Diencephalon. Two groups of neurons with contralateral projections.
(TXT)

Figure S6 Stable lines at various developmental stages and views for MEDMOD070042. Stage 32–34: Diencephalum pretectal nuclei, four rows of cells, two median two lateral row of cells. Medials are motoneurons, the lateral ones are sensory or interneurons.
(TXT)

Figure S7 Stable lines at various developmental stages and views for MEDMOD046007 and MEDMOD045693. MEDMOD046007 stage 32–34: Optic tectum (anterior and central zone), diencephalon and hypothalamus. MEDMOD045693 stage 24–32: Hypothalamus and maybe pectoral fins.
(TXT)

Figure S8 Stable lines at various developmental stages and views for MEDMOD086628. Stage 28–34: Rhombomeres.
(TXT)

Figure S9 Stable lines at various developmental stages and views for MEDMOD062537. Stage 22: Dorsal part of the retina and hypothalamus. Stage 25–32: Retina, forebrain and hypothalamus.
(TXT)

Table S1 List of all predicted CRMs. Medaka coordinates (genome version MEDAKA1) of all the predicted CRMs.
(PDF)

Table S2 List of predicted vertebrate conserved CRMs. Medaka coordinates (genome version MEDAKA1) of all the predicted vertebrate conserved CRMs.
(PDF)

Table S3 List of conserved and non-conserved CRMs for each tissue. Tab delimited list of tissues from ZFIN (column 1) with the corresponding medaka genes (column 2) and the corresponding CRM ids (column 3). The conservation of the CRMs is reported in column 4. For details, see the material and methods section.
(PDF)

Table S4 Enrichment of vertebrate conserved CRMs around genes expressed in neuronal tissues. For each selected developmental tissue (first column) and stage, the percentage of genes expressed in the given tissue that are linked to at least one vertebrate conserved CRMs (third column) or to at least one predicted CRMs (fourth column) is calculated. The statistical significance is calculated with a one-sided fisher test (second column). For details see methods section and Supplementary Table 3.
(PDF)

Table S5 Injection success rate. “Alive” column corresponds to the number of injected embryos which passed gastrulation. “Expression” corresponds to the number of embryos with expression pattern in the lens (successful injection) and “Specific Expression” corresponds to the number of embryos with reproducible expression pattern excluding the lens specific pattern.
(PDF)

Table S6 Genomic location, length (in bp), scores and enhancer activity of the tested CRMs. (**a**) For the 10 top scoring candidates. (**b**) For the 10 candidates evenly distributed amongst the 200 top scoring candidates.
(PDF)

Table S7 Candidates transcription factor predicted to bind CRMs. MEDMOD062537, MEDMOD045693 and MEDMOD086628. For each transcription factor, the name of the factor (from transfac or Jaspar), the name of the zebrafish homolog, the PWM and the partial expression pattern (from ZFIN) is recorded.
(PDF)

Table S8 Primer list. Description of all the primers used in this study (candidate CRMs cloning, *in-situ* probe generation).
(PDF)

## References

[1] Tsien RY (1998). The green fluorescent protein.. Annu Rev Biochem.

[2] Higashijima S, Masino MA, Mandel G, Fetcho JR (2003). Imaging neuronal activity during zebrafish behavior with a genetically encoded calcium indicator.. J Neurophysiol.

[3] Nagai T, Sawano A, Park ES, Miyawaki A (2001). Circularly permuted green fluorescent proteins engineered to sense Ca2+.. Proc Natl Acad Sci USA.

[4] Pertz O, Hodgson L, Klemke RL, Hahn KM (2006). Spatiotemporal dynamics of RhoA activity in migrating cells.. Nature.

[5] Srivastava J, Barber DL, Jacobson MP (2007). Intracellular pH sensors: design principles and functional significance.. Physiology (Bethesda).

[6] Heintz N (2001). BAC to the future: the use of bac transgenic mice for neuroscience research.. Nat Rev Neurosci.

[7] Parinov S, Kondrichin I, Korzh V, Emelyanov A (2004). Tol2 transposon-mediated enhancer trap to identify developmentally regulated zebrafish genes in vivo.. Dev Dyn.

[8] Ellingsen S, Laplante MA, Konig M, Kikuta H, Furmanek T (2005). Large-scale enhancer detection in the zebrafish genome.. Development.

[9] Korzh V (2007). Transposons as tools for enhancer trap screens in vertebrates.. Genome Biol.

[10] Scott EK, Mason L, Arrenberg AB, Ziv L, Gosse NJ (2007). Targeting neural circuitry in zebrafish using GAL4 enhancer trapping.. Nat Methods.

[11] Asakawa K, Suster ML, Mizusawa K, Nagayoshi S, Kotani T (2008). Genetic dissection of neural circuits by Tol2 transposon-mediated Gal4 gene and enhancer trapping in zebrafish.. Proc Natl Acad Sci USA.

[12] Pennacchio LA, Ahituv N, Moses AM, Prabhakar S, Nobrega MA (2006). In vivo enhancer analysis of human conserved non-coding sequences.. Nature.

[13] Fisher S, Grice EA, Vinton RM, Bessling SL, Urasaki A (2006). Evaluating the biological relevance of putative enhancers using Tol2 transposon-mediated transgenesis in zebrafish.. Nat Protoc.

[14] Woolfe A, Goodson M, Goode DK, Snell P, McEwen GK (2005). Highly conserved non-coding sequences are associated with vertebrate development.. PLoS Biol.

[15] Dermitzakis ET, Reymond A, Lyle R, Scamuffa N, Ucla C (2002). Numerous potentially functional but non-genic conserved sequences on human chromosome 21.. Nature.

[16] Bejerano G, Pheasant M, Makunin I, Stephen S, Kent WJ (2004). Ultraconserved elements in the human genome.. Science.

[17] Loots GG, Locksley RM, Blankespoor CM, Wang ZE, Miller W (2000). Identification of a coordinate regulator of interleukins 4, 13, and 5 by cross-species sequence comparisons.. Science.

[18] Lander ES, Linton LM, Birren B, Nusbaum C, Zody MC et al (2001). Initial sequencing and analysis of the human genome.. Nature.

[19] Waterston RH, Lindblad-Toh K, Birney E, Rogers J, Abril JF (2002). Initial sequencing and comparative analysis of the mouse genome.. Nature.

[20] International Chicken Genome Sequencing Consortium (2004). Sequence and comparative analysis of the chicken genome provide unique perspectives on vertebrate evolution.. Nature.

[21] Aparicio S, Chapman J, Stupka E, Putnam N, Chia J-M (2002). Whole-genome shotgun assembly and analysis of the genome of Fugu rubripes.. Science.

[22] Brudno M, Do CB, Cooper GM, Kim MF, Davydov E (2003). LAGAN and Multi-LAGAN: efficient tools for large-scale multiple alignment of genomic DNA.. Genome Res.

[23] Schwartz S, Kent WJ, Smit A, Zhang Z, Baertsch R (2003). Human-mouse alignments with BLASTZ.. Genome Res.

[24] Blanchette M, Kent WJ, Riemer C, Elnitski L, Smit AFA (2004). Aligning multiple genomic sequences with the threaded blockset aligner.. Genome Res.

[25] Paten B, Herrero J, Beal K, Birney E (2009). Sequence progressive alignment, a framework for practical large-scale probabilistic consistency alignment.. Bioinformatics.

[26] Howard ML, Davidson EH (2004). cis-Regulatory control circuits in development.. Dev Biol.

[27] Philippakis AA, He FS, Bulyk ML (2005). Modulefinder: a tool for computational discovery of cis regulatory modules.. Pac Symp Biocomput.

[28] Blanchette M, Bataille AR, Chen X, Poitras C, Laganiere J (2006). Genome-wide computational prediction of transcriptional regulatory modules reveals new insights into human gene expression.. Genome Res.

[29] Ferretti V, Poitras C, Bergeron D, Coulombe B, Robert F (2007). PReMod: a database of genome-wide mammalian cis-regulatory module predictions.. Nucleic Acids Res.

[30] Matys V, Kel-Margoulis OV, Fricke E, Liebich I, Land S (2006). TRANSFAC and its module TRANSCompel: transcriptional gene regulation in eukaryotes.. Nucleic Acids Res.

[31] Vlieghe D, Sandelin A, De Bleser PJ, Vleminckx K, Wasserman WW (2006). A new generation of JASPAR, the open-access repository for transcription factor binding site profiles.. Nucleic Acids Res.

[32] Ettwiller L, Paten B, Ramialison M, Birney E, Wittbrodt J (2007). Trawler: de novo regulatory motif discovery pipeline for chromatin immunoprecipitation.. Nat Methods.

[33] Haudry Y, Berube H, Letunic I, Weeber P-D, Gagneur J (2008). 4DXpress: a database for cross-species expression pattern comparisons.. Nucleic Acids Res.

[34] Sprague J, Bayraktaroglu L, Clements D, Conlin T, Fashena D (2006). The Zebrafish Information Network: the zebrafish model organism database.. Nucleic Acids Res.

[35] Grabher C, Wittbrodt J (2007). Meganuclease and transposon mediated transgenesis in medaka.. Genome Biol.

[36] Monteilhet C, Perrin A, Thierry A, Colleaux L, Dujon B (1990). Purification and characterization of the in vitro activity of I-Sce I, a novel and highly specific endonuclease encoded by a group I intron.. Nucleic Acids Res.

[37] Blechinger SR, Evans TG, Tang PT, Kuwada JY, Warren JT (2002). The heat-inducible zebrafish hsp70 gene is expressed during normal lens development under non-stress conditions.. Mech Dev.

[38] Smith B, Ashburner M, Rosse C, Bard J, Bug W (2007). The OBO Foundry: coordinated evolution of ontologies to support biomedical data integration.. Nat Biotechnol.

[39] Moens CB, Yan YL, Appel B, Force AG, Kimmel CB (1996). valentino: a zebrafish gene required for normal hindbrain segmentation.. Development.

[40] Cooke J, Moens C, Roth L, Durbin L, Shiomi K (2001). Eph signalling functions downstream of Val to regulate cell sorting and boundary formation in the caudal hindbrain.. Development.

[41] Rohrschneider MR, Elsen GE, Prince VE (2007). Zebrafish Hoxb1a regulates multiple downstream genes including prickle1b.. Dev Biol.

[42] Hernandez RE, Rikhof HA, Bachmann R, Moens CB (2004). vhnf1 integrates global RA patterning and local FGF signals to direct posterior hindbrain development in zebrafish.. Development.

[43] Nakai S, Kawano H, Yudate T, Nishi M, Kuno J (1995). The POU domain transcription factor Brn-2 is required for the determination of specific neuronal lineages in the hypothalamus of the mouse.. Genes Dev.

[44] Blow MJ, McCulley DJ, Li Z, Zhang T, Akiyama JA (2010). ChIP-Seq identification of weakly conserved heart enhancers.. Nat Genet.

[45] Flicek P, Aken BL, Beal K, Ballester B, Caccamo M (2008). Ensembl 2008.. Nucleic Acids Res.

[46] Kasahara M, Naruse K, Sasaki S, Nakatani Y, Qu W (2007). The medaka draft genome and insights into vertebrate genome evolution.. Nature.

[47] Jaillon O, Aury J-M, Brunet F, Petit J-L, Stange-Thomann N (2004). Genome duplication in the teleost fish Tetraodon nigroviridis reveals the early vertebrate proto-karyotype.. Nature.

[48] Kasprzyk A, Keefe D, Smedley D, London D, Spooner W (2004). EnsMart: a generic system for fast and flexible access to biological data.. Genome Res.

[49] Rembold M, Lahiri K, Foulkes NS, Wittbrodt J (2006). Transgenesis in fish: efficient selection of transgenic fish by co-injection with a fluorescent reporter construct.. Nat Protoc.

[50] Iwamatsu T (2004). Stages of normal development in the medaka Oryzias latipes.. Mech Dev.

[51] Loosli F, Winkler S, Burgtorf C, Wurmbach E, Ansorge W (2001). Medaka eyeless is the key factor linking retinal determination and eye growth.. Development.

