# Supplementary figures and images for: Combining Computational Prediction of Cis-Regulatory Elements with a New Enhancer Assay to Efficiently Label Neuronal Structures in the Medaka Fish
Source: PLoS One. 2011 May 27;6(5):e19747. doi: 10.1371/journal.pone.0019747 (PMC3103512; doi:10.1371/journal.pone.0019747)

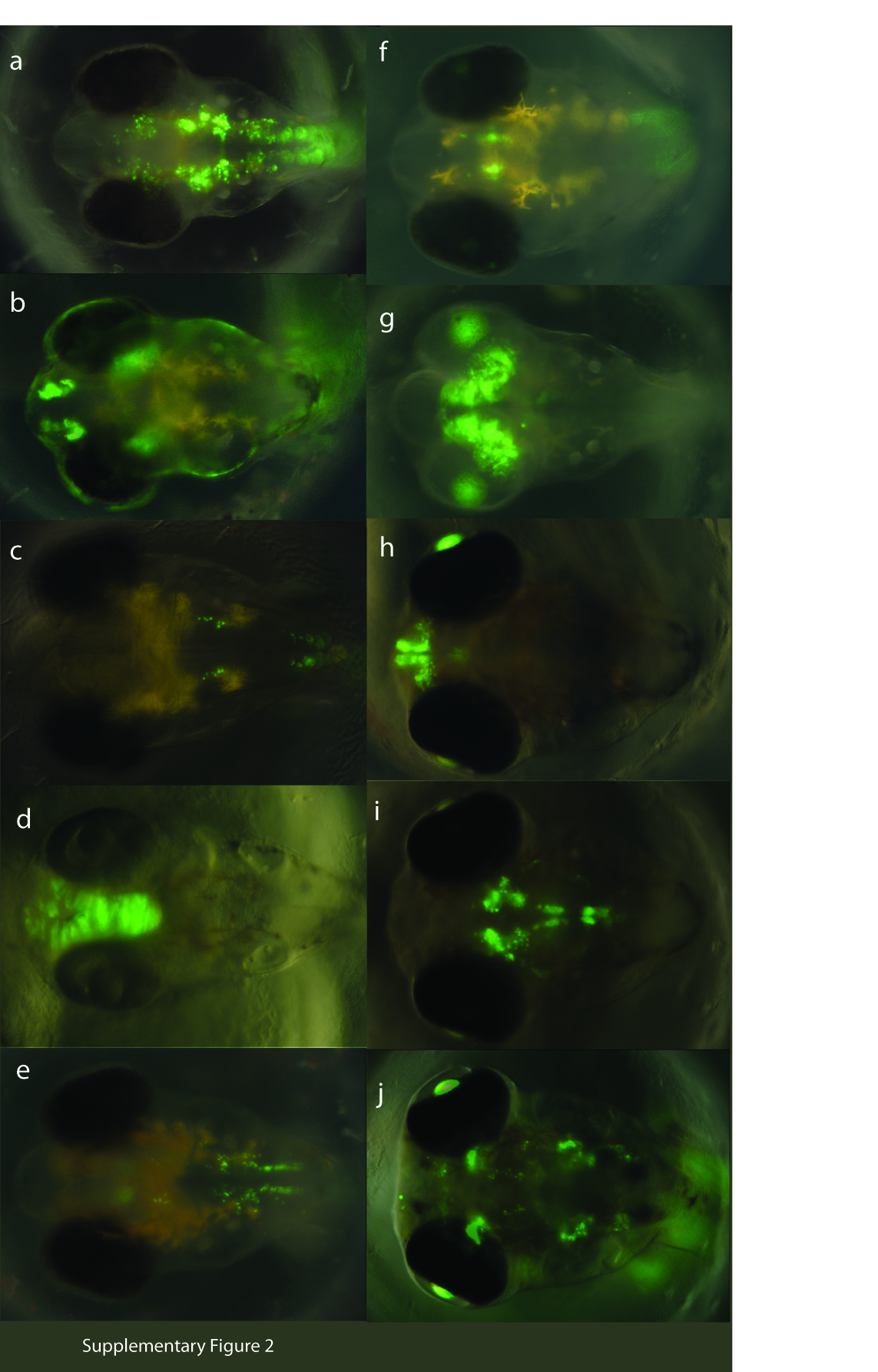

Supplement: Figure S2 — Enhancer activity of the additional 10 predicted CRMs evenly distributed among the 200 top scoring candidates. Example of injected fish showing a reproducible expression pattern. (a) MEDMOD021445 (b) MEDMOD092210 (c) MEDMOD062490 (d) MEDMOD057815 (e) MEDMOD021442 (f) MEDMOD093196 (g) MEDMOD062408 (h) MEDMOD047799 (i) MEDMOD083481 (j) MEDMOD062206. (TXT) [file pone.0019747.s002.txt]

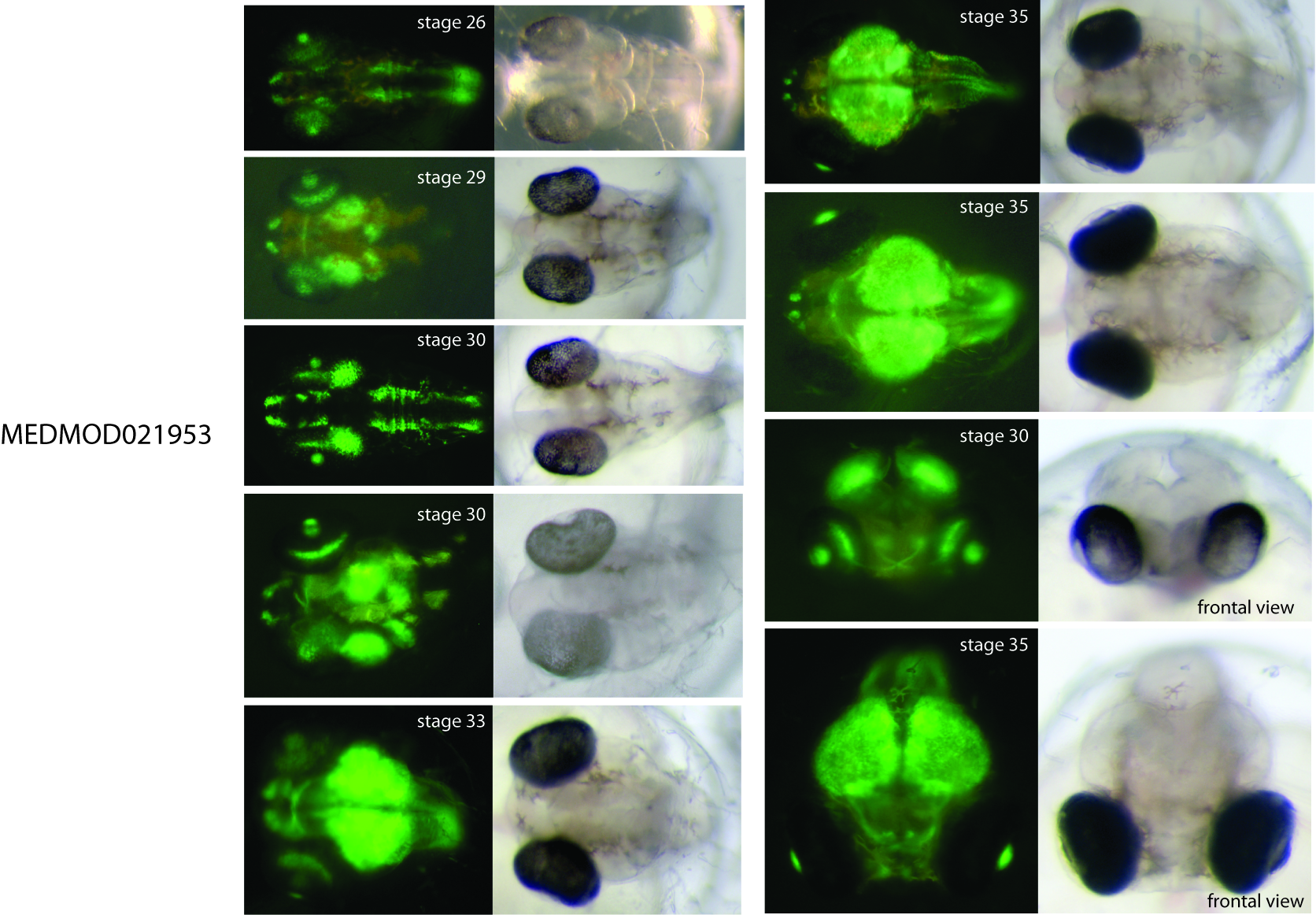

Supplement: Figure S3 — Stable lines at various developmental stages and views for MEDMOD021953. Stage 26–30: Telencephalon, retina ganglion cells (RGCs), tectum central zone, cerebellum, hypothalamus, rombomeres, alar plate. Stage 33–35: Telencephalon, tectum central zone, hypothalamus, cerebellum, hindbrain, RGCs, tegmentum. (TXT) [file pone.0019747.s003.txt]

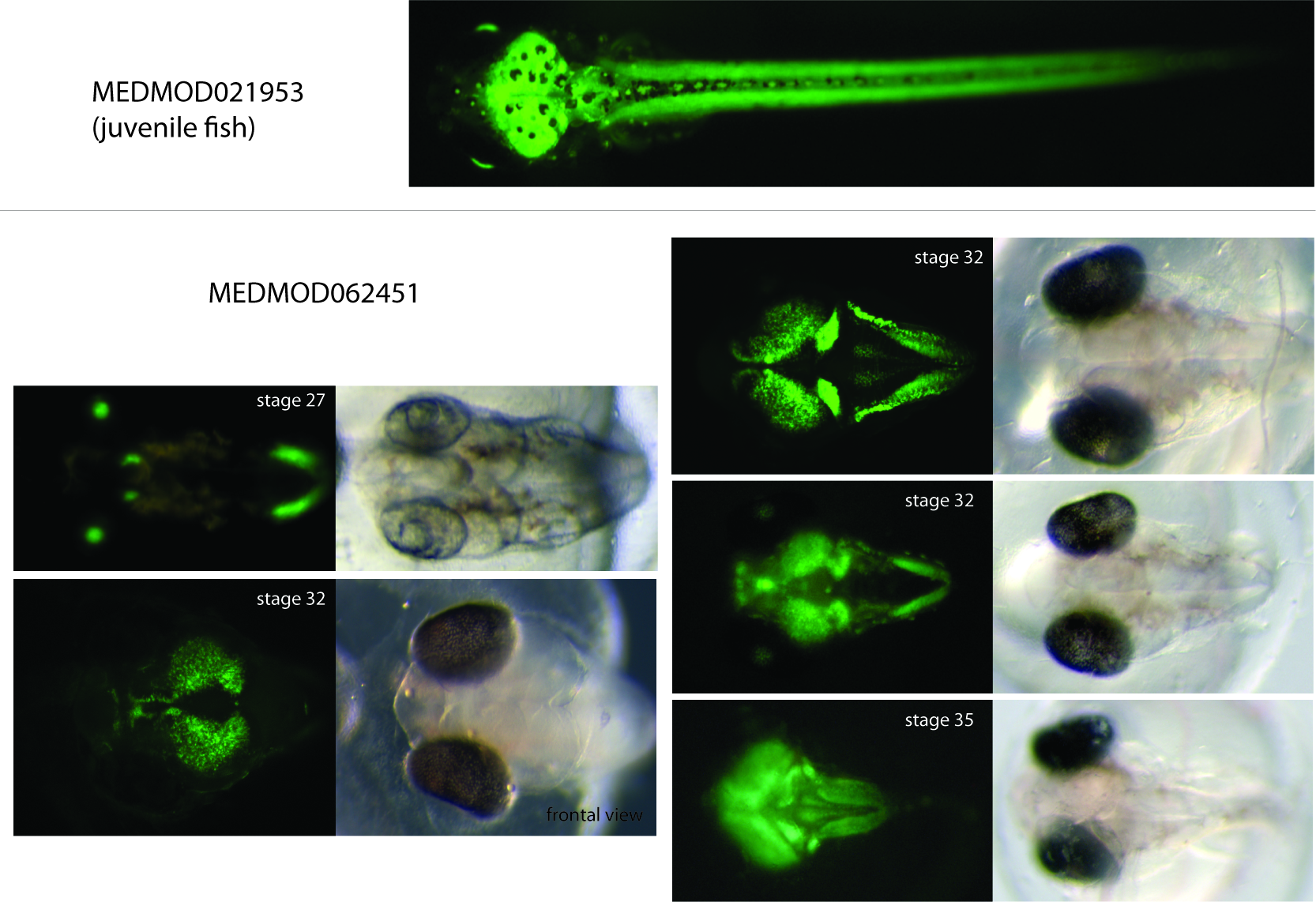

Supplement: Figure S4 — Stable lines at various developmental stages and views for MEDMOD062451. Stage 27: One group of cells in the hypothalamus (bilateral) or tegmentum. Stage 32: Optic tectum differentiated cells (central zone), torsus semicircularis, few cells in cerebellum, lateral part of the myelencephalon, hindbrain: two lateral rows cells and two medial rows of cells (motor neurons). Stage 35: Telencephalon posterior (area ventro-posterior), optic tectum: differentiated cells (peri-ventricular grey zone, deap layer), torsus semicircularis, midbrain dorsal, a few cells in the cerebellum anlage, hindbrain: Two lateral rows cells and two medial rows of cells (motoneurons). (TXT) [file pone.0019747.s004.txt]

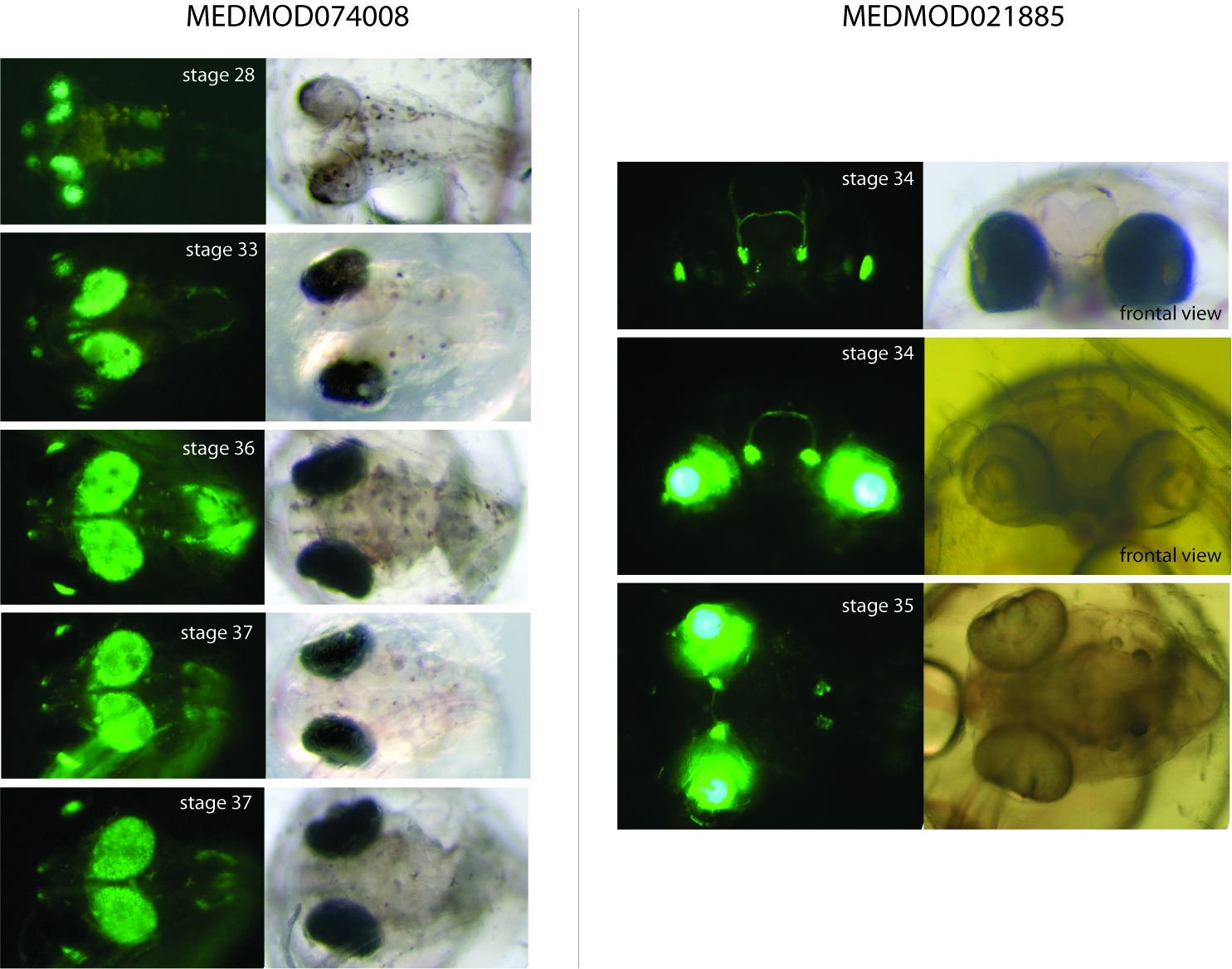

Supplement: Figure S5 — Stable lines at various developmental stages and views for MEDMOD074008 and MEDMOD021885. MEDMOD074008 stages 28–37: Tectum central zone and olfactory pits. MEDMOD021885 stages 34–35: Diencephalon. Two groups of neurons with contralateral projections. (TXT) [file pone.0019747.s005.txt]

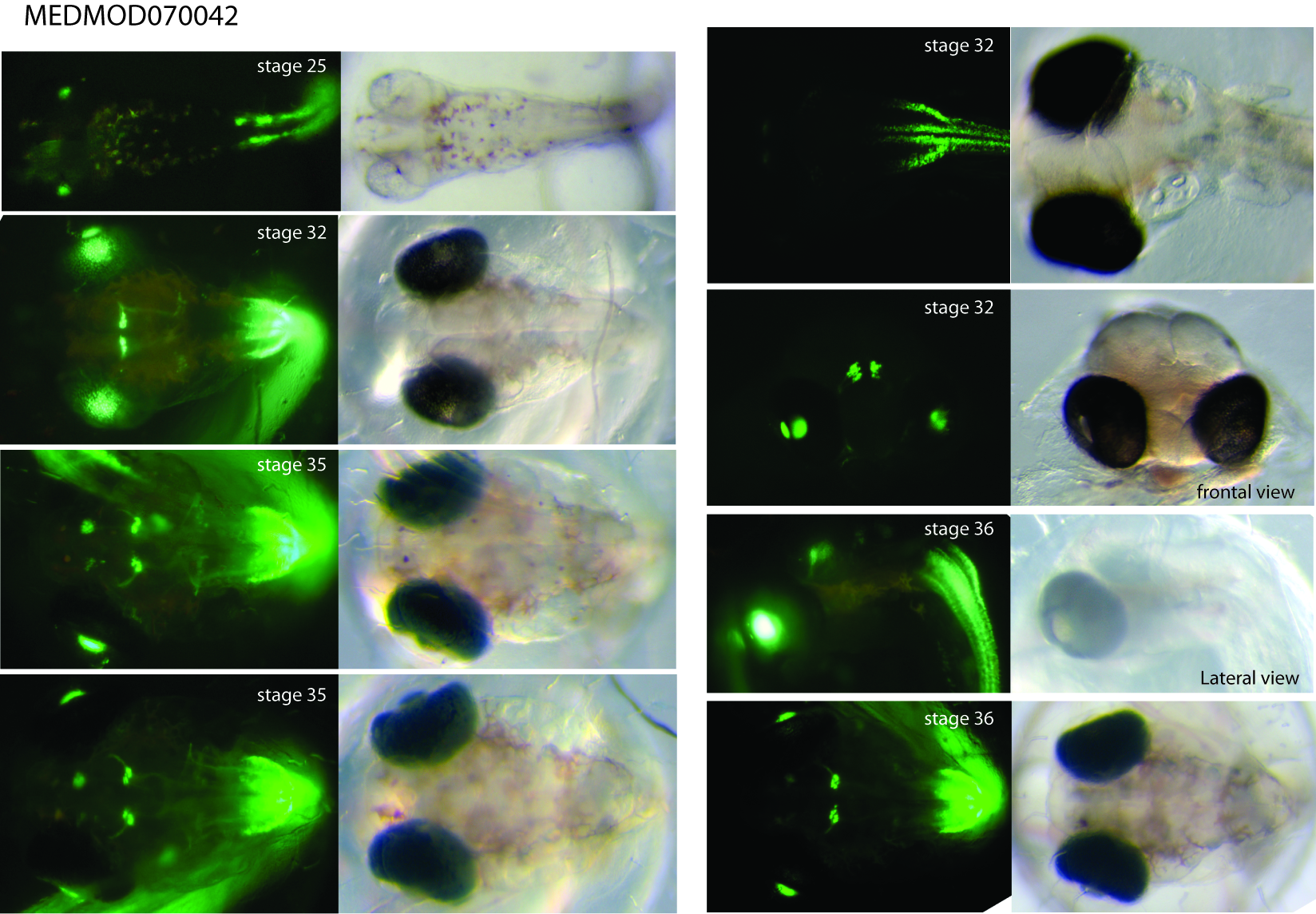

Supplement: Figure S6 — Stable lines at various developmental stages and views for MEDMOD070042. Stage 32–34: Diencephalum pretectal nuclei, four rows of cells, two median two lateral row of cells. Medials are motoneurons, the lateral ones are sensory or interneurons. (TXT) [file pone.0019747.s006.txt]

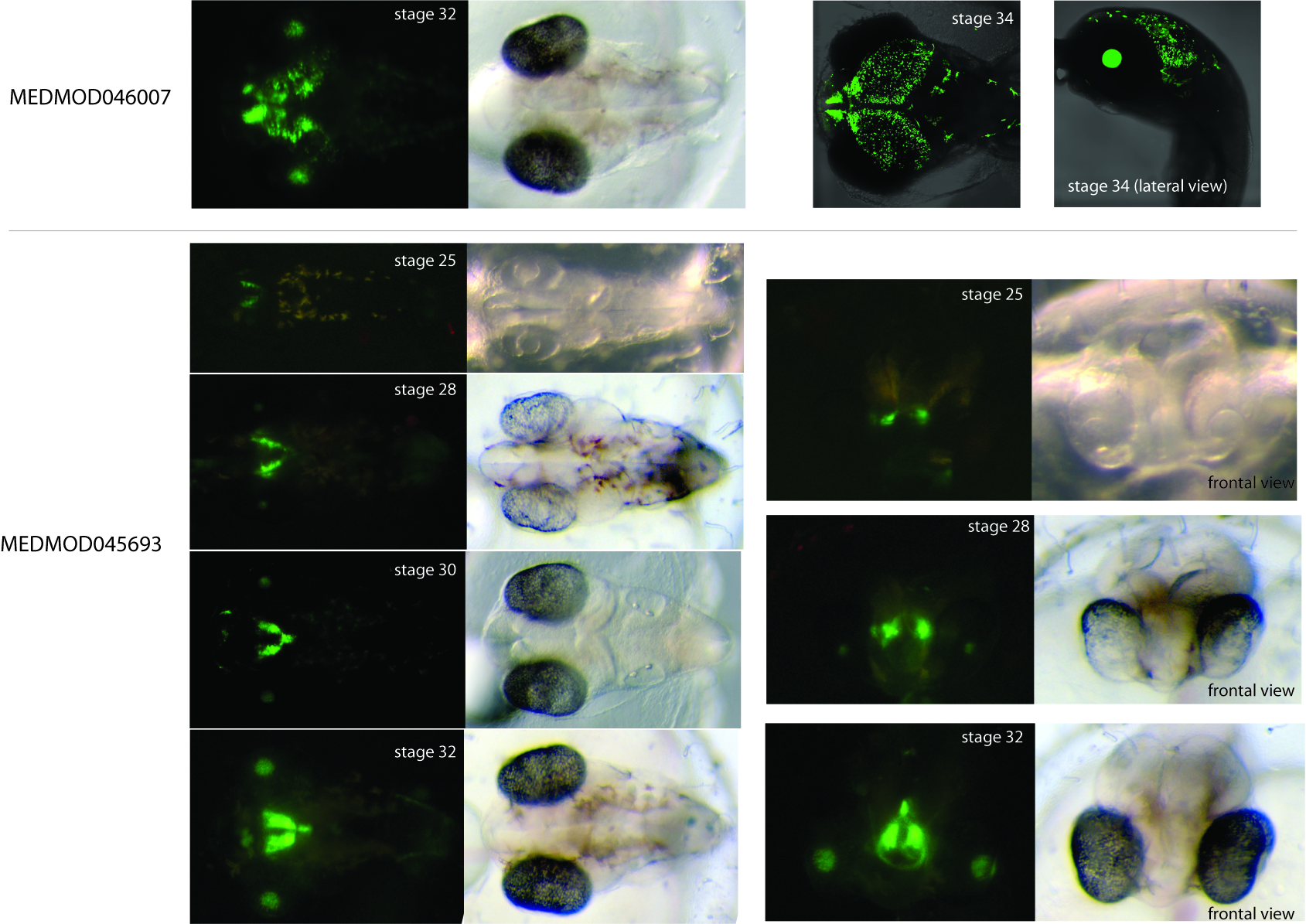

Supplement: Figure S7 — Stable lines at various developmental stages and views for MEDMOD046007 and MEDMOD045693. MEDMOD046007 stage 32–34: Optic tectum (anterior and central zone), diencephalon and hypothalamus. MEDMOD045693 stage 24–32: Hypothalamus and maybe pectoral fins. (TXT) [file pone.0019747.s007.txt]

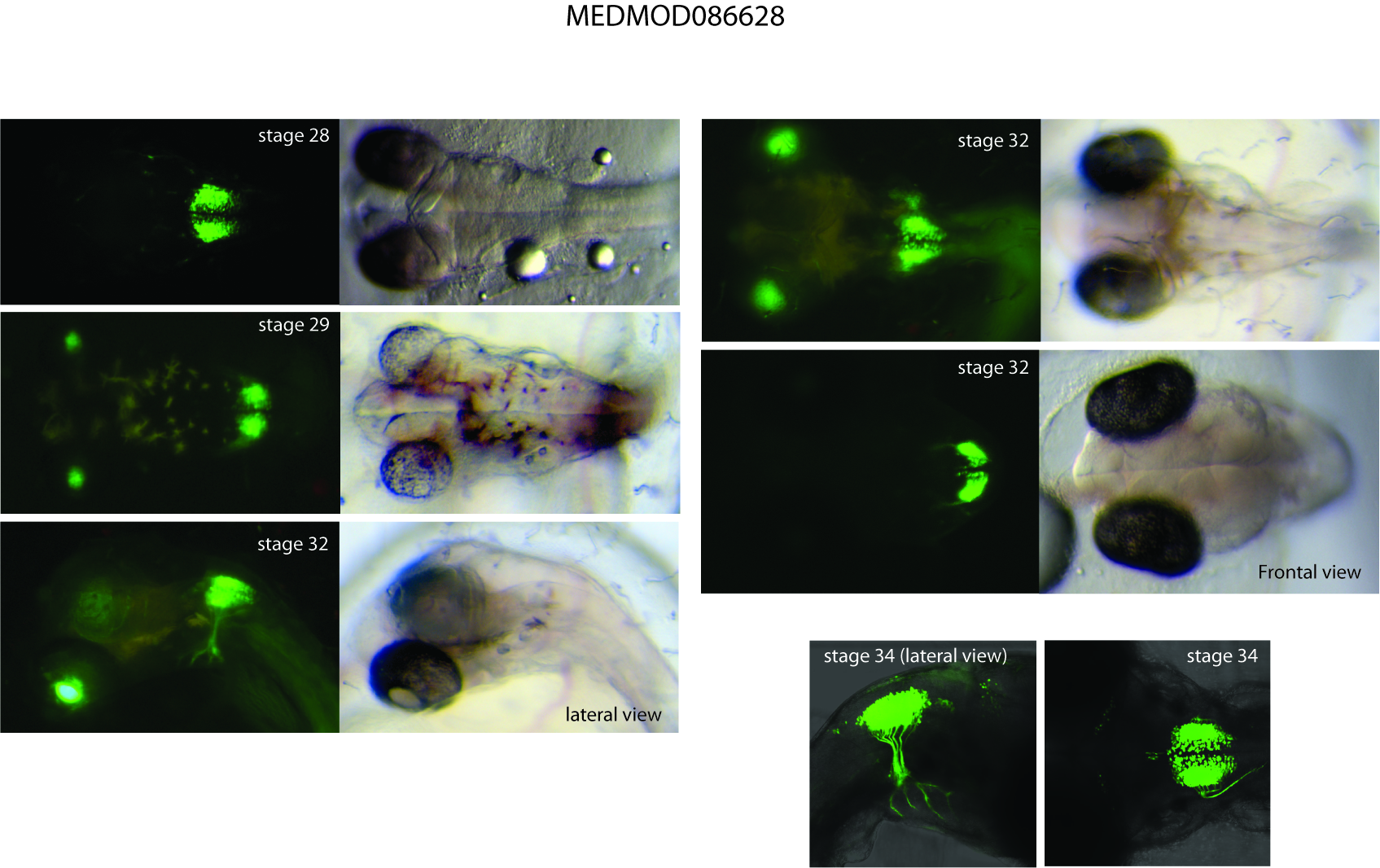

Supplement: Figure S8 — Stable lines at various developmental stages and views for MEDMOD086628. Stage 28–34: Rhombomeres. (TXT) [file pone.0019747.s008.txt]

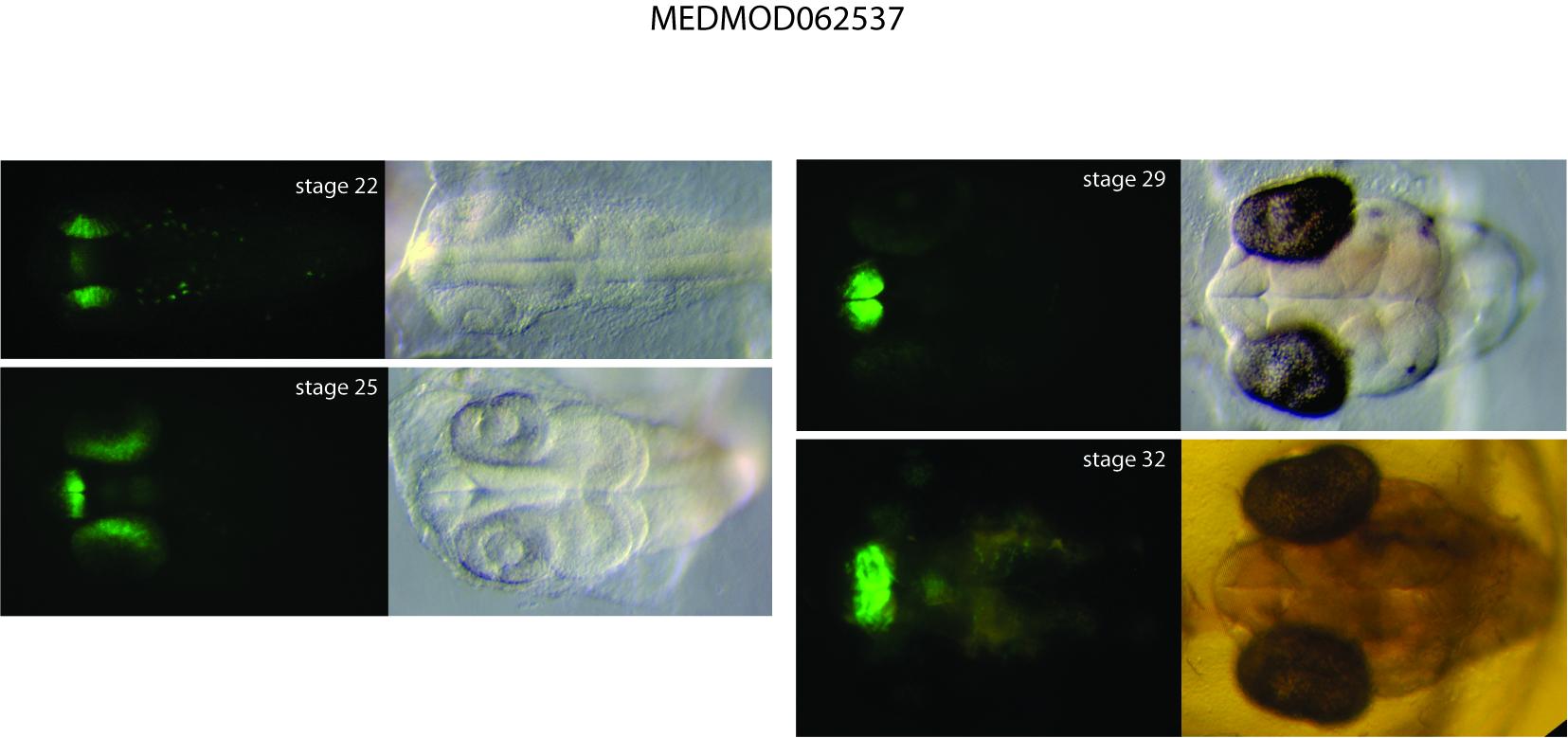

Supplement: Figure S9 — Stable lines at various developmental stages and views for MEDMOD062537. Stage 22: Dorsal part of the retina and hypothalamus. Stage 25–32: Retina, forebrain and hypothalamus. (TXT) [file pone.0019747.s009.txt]
