# Supplementary material for: Combining Computational Prediction of Cis-Regulatory Elements with a New Enhancer Assay to Efficiently Label Neuronal Structures in the Medaka Fish
Source: PLoS One. 2011 May 27;6(5):e19747. doi: 10.1371/journal.pone.0019747 (PMC3103512; doi:10.1371/journal.pone.0019747)
Supplement: Table S4 — Enrichment of vertebrate conserved CRMs around genes expressed in neuronal tissues. For each selected developmental tissue (first column) and stage, the percentage of genes expressed in the given tissue that are linked to at least one vertebrate conserved CRMs (third column) or to at least one predicted CRMs (fourth column) is calculated. The statistical significance is calculated with a one-sided fisher test (second column). For details see methods section and Supplementary Table 3. (PDF) [file pone.0019747.s013.pdf]

Tissue	p-values	% of genes expressed in tissue linked to human conserved set	% of genes expressed in tissue linked to background
midbrain hindbrain boundary	2.94E-05	20.95	10.99
hindbrain	0.0002373	55.71	43.25
brain	0.0005317	74.29	63.2
diencephalon	0.000836	53.81	42.59
neural rod	0.001223	24.29	15.84
central nervous system	0.001814	76.19	66.6
spinal cord	0.002601	42.86	33.21
presumptive diencephalon	0.003885	10.48	5.533
neural keel	0.004098	34.29	25.71
neural tube	0.004157	42.86	33.72
hypothalamus	0.004558	14.29	8.544
midbrain	0.006946	53.33	44.48
pectoral fin bud	0.01151	10.48	6.116
pectoral fin	0.01156	26.19	19.44
optic tectum	0.01301	36.67	29.19
forebrain	0.01667	57.62	49.92
neural plate	0.02073	31.9	25.32
otic vesicle	0.0292	20	14.88
cerebellum	0.04099	14.76	10.62
anterior neural keel	0.05104	22.38	17.7
retinal ganglion cell layer	0.05531	14.76	10.92
retinal neural layer	0.0613	20.95	16.60
anterior neural rod	0.06567	10	6.967
alar plate midbrain	0.06612	10.95	7.787
tegmentum	0.0667	24.76	20.22
ectoderm	0.08338	21.43	17.45
midbrain neural keel	0.1047	15.71	12.52
nervous system	0.1471	17.62	14.75
midbrain neural tube	0.1641	20	17.17
hindbrain neural plate	0.1659	10.95	8.780
optic vesicle	0.1706	15.71	13.21
posterior neural plate	0.1805	10.95	8.890
neuron	0.1815	15.24	12.86
anterior neural tube	0.1991	20	17.51
vestibuloauditory system	0.2206	11.9	10.06
pronephros	0.2261	16.67	14.58
retina	0.2342	35.71	33.09
pronephric duct	0.2681	11.9	10.37
telencephalon	0.2686	33.33	31.10
epithalamus	0.2814	12.38	10.91
immature eye	0.3067	25.24	23.52
lateral line system	0.3926	12.86	12.04
renal system	0.4163	17.62	16.87
visual system	0.4214	45.24	44.31
pharyngeal arch	0.4331	22.38	21.69
somite	0.4568	21.43	20.92
eye	0.4731	42.38	41.91
cranium	0.4779	17.62	17.28
peripheral nervous system	0.4981	12.86	12.66
basal plate midbrain	0.5156	11.9	11.81
ventricular zone	0.5807	10	10.25
ventricular system	0.585	10.95	11.24
pharyngeal arch 3-7 skeleton	0.6492	10.95	11.62
cardiovascular system	0.6713	11.43	12.25
pharyngeal arch 3-7	0.677	10.95	11.79
notochord	0.7396	14.76	16.20
trunk	0.8863	39.05	42.99
head	0.9579	53.81	59.54
mesoderm	0.9594	23.81	29.04
whole organism	0.9922	55.71	63.71
digestive system	0.9971	16.19	23.88
endocrine system	0.9987	12.38	20.13
