# Supplementary material for: Combining Computational Prediction of Cis-Regulatory Elements with a New Enhancer Assay to Efficiently Label Neuronal Structures in the Medaka Fish
Source: PLoS One. 2011 May 27;6(5):e19747. doi: 10.1371/journal.pone.0019747 (PMC3103512; doi:10.1371/journal.pone.0019747)
Supplement: Table S5 — Injection success rate. “Alive” column corresponds to the number of injected embryos which passed gastrulation. “Expression” corresponds to the number of embryos with expression pattern in the lens (successful injection) and “Specific Expression” corresponds to the number of embryos with reproducible expression pattern excluding the lens specific pattern. (PDF) [file pone.0019747.s014.pdf]

Supplementary Table S5

Constructs	Injected Eggs	Alive	Expression (lens)	Specific Expression	Percentage of Expression	Percentage of Specific Expression
hsp70	259	189	101	101	53.44	100.00
MEDMOD021953-hsp70::GFP	178	84	32	20	38.10	62.50
MEDMOD062451-hsp70::GFP	294	233	67	48	28.76	71.64
MEDMOD074008-hsp70::GFP	157	111	53	35	47.75	66.04
MEDMOD021885-hsp70::GFP	297	249	84	27	33.73	32.14
MEDMOD070042-hsp70::GFP	166	139	65	36	46.76	55.38
MEDMOD046007-hsp70::GFP	167	119	43	20	36.13	46.51
MEDMOD046561-hsp70::GFP	74	69	32	0	46.38	0.00
MEDMOD045693-hsp70::GFP	180	88	19	12	21.59	63.16
MEDMOD086628-hsp70::GFP	97	76	35	26	46.05	74.29
MEDMOD062537-hsp70::GFP	205	151	56	32	37.09	57.14
MEDMOD021445-hsp70::GFP	79	74	43	27	58.11	62.79
MEDMOD092210-hsp70::GFP	82	61	34	20	55.74	58.82
MEDMOD062490-hsp70::GFP	107	87	65	37	74.71	56.92
MEDMOD057815-hsp70::GFP	49	45	23	19	51.11	82.61
MEDMOD021442-hsp70::GFP	60	48	17	14	35.42	82.35
MEDMOD093196-hsp70::GFP	39	35	12	10	34.29	83.33
MEDMOD062408-hsp70::GFP	104	79	49	38	62.03	77.55
MEDMOD047799-hsp70::GFP	65	49	29	28	59.18	96.55
MEDMOD083481-hsp70::GFP	75	64	32	23	50.00	71.88
MEDMOD062206-hsp70::GFP	72	68	44	25	64.71	56.82
