# Supplementary material for: Combining Computational Prediction of Cis-Regulatory Elements with a New Enhancer Assay to Efficiently Label Neuronal Structures in the Medaka Fish
Source: PLoS One. 2011 May 27;6(5):e19747. doi: 10.1371/journal.pone.0019747 (PMC3103512; doi:10.1371/journal.pone.0019747)
Supplement: Table S6 — Genomic location, length (in bp), scores and enhancer activity of the tested CRMs. (a) For the 10 top scoring candidates. (b) For the 10 candidates evenly distributed amongst the 200 top scoring candidates. (PDF) [file pone.0019747.s015.pdf]

Supplementary Table S6

a

position in the list	id	chromosome	start	end	length (in bp)	score	enhancer activity
#1	MEDMOD021953	chr15	18873216	18876120	2904	61.5114	+
#2	MEDMOD062451	chr3	18031644	18032442	798	60.9452	+
#3	MEDMOD074008	chr6	12052186	12052995	809	58.5236	+
#4	MEDMOD021885	chr15	18847231	18848265	1034	54.7302	+
#5	MEDMOD070042	chr4	17303490	17304535	1045	54.4804	+
#7	MEDMOD046007	chr21	23236651	23238901	2250	44.4189	+
#8	MEDMOD046561	chr21	24507548	24508362	814	43.5749	–
#9	MEDMOD045693	chr21	22539537	22540529	992	43.2247	+
#10	MEDMOD086628	chr9	6167346	6167743	397	42.9471	+
#11	MEDMOD062537	chr3	18144435	18145588	1153	42.3505	+
**b**							
#20	MEDMOD021445	chr15	18115289	18116010	721	40.1251	+
#40	MEDMOD092210	chr5	4978486	4979194	708	35.4542	+
#60	MEDMOD062490	chr3	18079296	18080073	777	30.9411	+
#81	MEDMOD057815	chr24	7038192	7039261	1069	29.3093	+
#100	MEDMOD021442	chr15	18108429	18109084	655	26.8599	+
#120	MEDMOD093196	chr5	10470136	10470700	564	24.7016	+
#140	MEDMOD062408	chr3	17981288	17981793	505	22.9661	+
#159	MEDMOD047799	chr22	192677	193287	610	22.0017	+
#180	MEDMOD083481	chr8	7484395	7484907	512	20.785	+
#200	MEDMOD062206	chr3	17606719	17607226	507	19.692	+
