# Supplementary material for: Combining Computational Prediction of Cis-Regulatory Elements with a New Enhancer Assay to Efficiently Label Neuronal Structures in the Medaka Fish
Source: PLoS One. 2011 May 27;6(5):e19747. doi: 10.1371/journal.pone.0019747 (PMC3103512; doi:10.1371/journal.pone.0019747)
Supplement: Table S7 — Candidates transcription factor predicted to bind CRMs. MEDMOD062537, MEDMOD045693 and MEDMOD086628. For each transcription factor, the name of the factor (from transfac or Jaspar), the name of the zebrafish homolog, the PWM and the partial expression pattern (from ZFIN) is recorded. (PDF) [file pone.0019747.s016.pdf]

	Factor name (premod)	factor name (Danio rerio)	PWM	Zfin Annotation (Curated)
**MEDMOD062537**	foxd1	foxd1	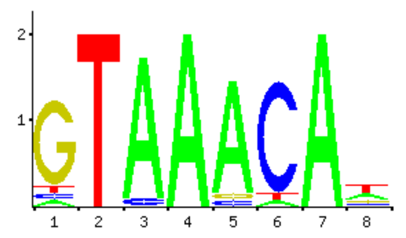	Forebrain and more
	sox2	Sox2	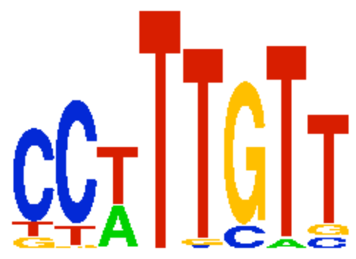	Forebrain and more
	sox9	Sox9b	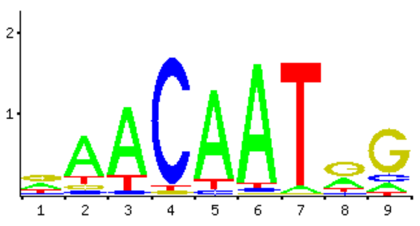	Forebrain and more
	myc	myca	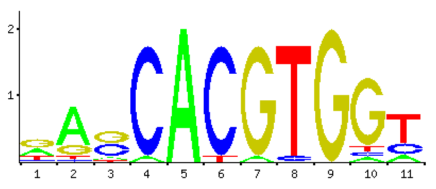	Forebrain and more
**MEDMOD045693**	pou3f2	pou3f2	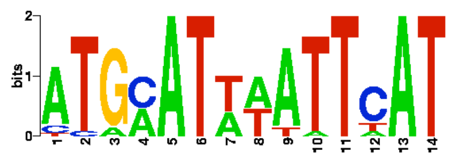	diencephalon and more structures
	dl	dla	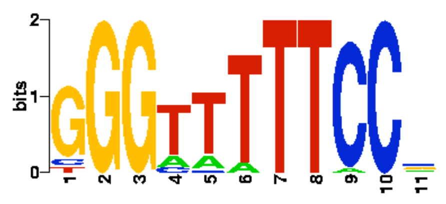	diencephalon and more structures
	dl	dlb	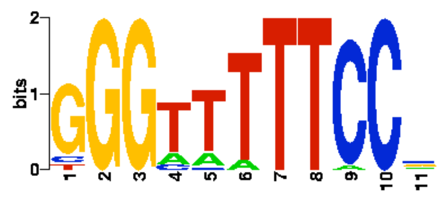	diencephalon and more structures
	HNF-6	onecut1	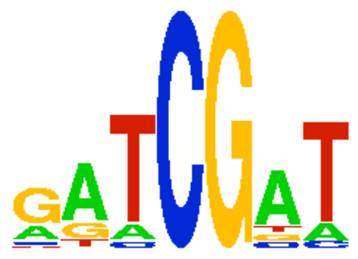	diencephalon and more structures
	Fos	Fos	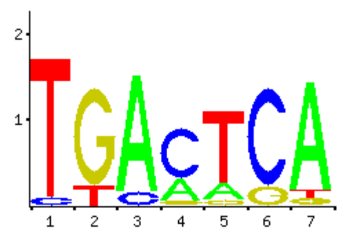	diencephalon and more structures
**MEDMOD086628**	Mafb	Mafba (mal)	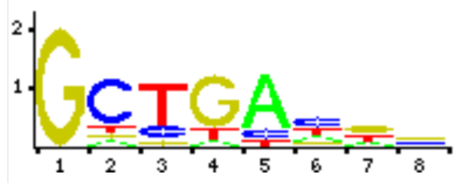	Rhombomeres only
	Mef2a	mef2a	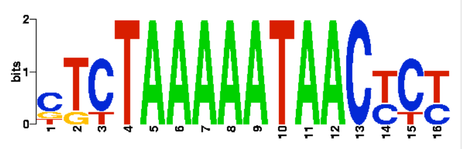	Hindbrain and more
	sox9	sox9b	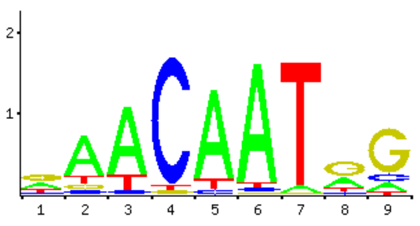	Rhombomeres and more
	Evi1	mecom	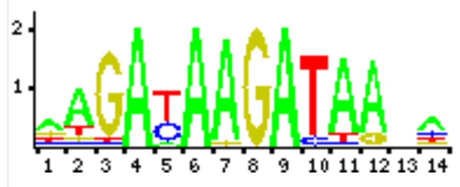	Hindbrain and more
	Elf1	efna2	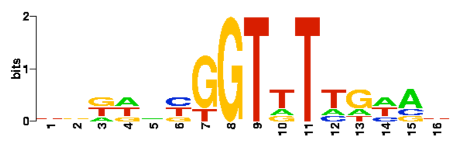	Hindbrain and more
	En1	Eng1b	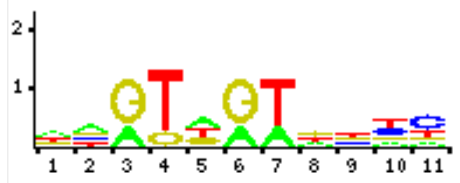	Hindbrain and more
	Lhx3	Lhx3	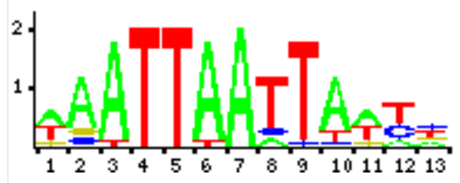	Hindbrain and more
	Msx1	Msxe	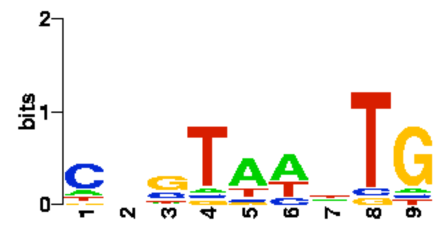	Hindbrain and more
