# Supplementary material for: Combining Computational Prediction of Cis-Regulatory Elements with a New Enhancer Assay to Efficiently Label Neuronal Structures in the Medaka Fish
Source: PLoS One. 2011 May 27;6(5):e19747. doi: 10.1371/journal.pone.0019747 (PMC3103512; doi:10.1371/journal.pone.0019747)
Supplement: Table S8 — Primer list. Description of all the primers used in this study (candidate CRMs cloning, in-situ probe generation). (PDF) [file pone.0019747.s017.pdf]

Supplementary Table S8

cloning of the top 10 predicted CRMs

primer ID	Primer sequences	position in the list
MEDMOD021953_fwd	GACCTTAAGCTTACATGCACTCCCCCTGTTAAAGCCC	#1
MEDMOD021953_rev	GACCTTAAGCTTTAGTTTTTGACAGCATCATAATAATC	#1
MEDMOD062451_fwd	GACCTTAAGCTTACCATATAGGAAGTAATGAAACCTG	#2
MEDMOD062451_rev	GACCTTAAGCTTAGTGGTTACATCTGCTCCATTCTTTC	#2
MEDMOD074008_fwd	GACCTTAAGCTTATTCATACTTTTGCTTTTTCCCTTTT	#3
MEDMOD074008_rev	GACCTTAAGCTTCCTTAAACCAACTATAGTAAACTGGC	#3
MEDMOD021885_fwd	GACCTTAAGCTTGAATCTGCTAGAGTTTACTTTACAA	#4
MEDMOD021885_rev	GACCTTAAGCTTCTGCCTGACTATTGCCTCATTGGC	#4
MEDMOD070042_fwd	GACCTTAAGCTTTCCCTGAACTCATATATATACACACAC	#5
MEDMOD070042_rev	GACCTTAAGCTTAAATACATTAGAAGAAGGAAGACTG	#5
MEDMOD046007_fwd	GACCTTAAGCTTCTAAGGACCAGGACAAGTTGATTGCC	#7
MEDMOD046007_rev	GACCTTAAGCTTAACCTCTGTGCATTGTTGTAATTCTA	#7
MEDMOD046561_fwd	GACCTTAAGCTTTGGCTGCCCAATTGTTGGTTTTACG	#8
MEDMOD046561_rev	GACCTTAAGCTTCCGCTAACCGCTTCCCTACAGGA	#8
MEDMOD045693_fwd	GACCTTAAGCTTGATAATTGACACAGTCTATCATTGC	#9
MEDMOD045693_rev	GACCTTAAGCTTTGTTATTCTAAATCAAAACCATAC	#9
MEDMOD086628_fwd	GACCTTAAGCTTCAAAAGCATTCTGTTGTATTCCCCTG	#10
MEDMOD086628_rev	GACCTTAAGCTTCCACCCTTCCCATAAGTGCCCCAGCT	#10
MEDMOD062537_fwd	GACCTTAAGCTTGGCCAAAAAGAAGTGCCATAATGAT	#11
MEDMOD062537_rev	GACCTTAAGCTTAAGCAGGAGCTGAAGCAGGCAATTGG	#11

cloning of the 10 predicted CRMs evenly distributed amongst the 200 top candidate

primer ID	Primer sequences	position in the list
MEDMOD021445_fwd	GACCTTAAGCTTTTCGTGACAAAAGTTTGG	#20
MEDMOD021445_rev	GACCTTAAGCTTAATTACTAGAGAAAATG	#20
MEDMOD092210_fwd	GACCTTAAGCTTGTCCTTGATGTGTGTTAAATGG	#40
MEDMOD092210_rev	GACCTTAAGCTTCTACTGAGACAGCAAGGACACC	#40
MEDMOD062490_fwd	GACCTTAAGCTTTACAGTTTCTCGTAAG	#60
MEDMOD062490_rev	GACCTTAAGCTTTGGCACATGAATCGCG	#60
MEDMOD057815_fwd	GACCTTAAGCTTTGGCTCGATTTGTCAAC	#81
MEDMOD057815_rev	GACCTTAAGCTTAGAACTGATGAAAGCAG	#81
MEDMOD021442_fwd	GACCTTAAGCTTAATCTTTTGTCCAACATCGC	#100
MEDMOD021442_rev	GACCTTAAGCTTAACATGACAACATAATAGGC	#100
MEDMOD093196_fwd	GACCTTAAGCTTTTTCAAACTGTCCTTTAAGC	#120
MEDMOD093196_rev	GACCTTAAGCTTGCGTTGTTTAACACTACC	#120
MEDMOD062408_fwd	GACCTTAAGCTTTATCTATACTTCACTGTC	#140
MEDMOD062408_rev	GACCTTAAGCTTTTTGAGAATGCTACTGCG	#140
MEDMOD047799_fwd	GACCTTAAGCTTGGCTGCAATCACTTTCAG	#159
MEDMOD047799_rev	GACCTTAAGCTTCGCCGGCCCCTCGCCCC	#159
MEDMOD083481_fwd	GACCTTAAGCTTCGCAGCTTTGTTTATATCGG	#180
MEDMOD083481_rev	GACCTTAAGCTTAGCTGCATTGATTTGACAGC	#180
MEDMOD062206_fwd	GACCTTAAGCTTATAAAATACAATATTGTC	#200
MEDMOD062206_rev	GACCTTAAGCTTAAATTTGAAAAACAAAAGC	#200

Primer sequences for the in-situs:

ATG4C_fwd: GCAGTGTGTCTGAAGCTGG
ATG4C_rev: GCTCAGAATGTTGCCTGTC
GON3_ORYLA_fwd: CTAATGGACGTGAGCAGCAAAG
GON3_ORYLA_rev: GCTTTGTTAGTTGTGATTTCTTTTT
KCNH7_fwd: TACTGCAACGATGGCTTCTG
KCNH7_rev: TGTGCGTTCTGTCTTTGACC
FIGN (1 of 2)_fwd: CTAGGTCTAAAGATGCAGTGGAC
FIGN (1 of 2)_rev: AGTGCAGGTAGGAGCCGTTG
SCAMP1_fwd: GTTCATCACGCATCCTCTCATC
SCAMP1_rev: CACGGGACGCATCTACAATAAAC

green : additional sequences
red : cloning site
